# *In Vitro* Adherence of Oral Bacteria to Different Types of Tongue Piercings

**DOI:** 10.1155/2016/7349371

**Published:** 2016-09-20

**Authors:** Lucas Pereira Borges, Julio Cesar Campos Ferreira-Filho, Julia Medeiros Martins, Caroline Vieira Alves, Bianca Marques Santiago, Ana Maria Gondim Valença

**Affiliations:** ^1^Department of Clinic and Social Dentistry, School of Dentistry, Universidade Federal da Paraíba (UFPB), Campus I, Jardim Cidade Universitária, 58051-900 João Pessoa, PB, Brazil; ^2^Department of Pediatric Dentistry and Orthodontics, School of Dentistry, Universidade Federal do Rio de Janeiro (UFPB), 255 Rodolpho Paulo Rocco Street, 21941-913 Rio de Janeiro, RJ, Brazil

## Abstract

The purpose of this work was to verify* in vitro* adherence of* E. corrodens* and* S. oralis* to the surface of tongue piercings made of surgical steel, titanium, Bioplast, and Teflon. For this, 160 piercings were used for the count of Colony Forming Units (CFU) and 32 piercings for analysis under scanning electron microscopy. Of these, 96 (24 of each type) were individually incubated in 5 mL of BHI broth and 50 *μ*L of inoculum at 37°C/24 h. The other 96 piercings formed the control group and were individually incubated in 5 mL of BHI broth at 37°C/24 h. Plates were incubated at 37°C/48 h for counting of CFU/mL and data were submitted to statistical analysis (*p* value <0.05). For* E. corrodens*, difference among types of material was observed (*p* < 0.001) and titanium and surgical steel showed lower bacterial adherence. The adherence of* S. oralis* differed among piercings, showing lower colonization (*p* < 0.007) in titanium and surgical steel piercings. The four types of piercings were susceptible to colonization by* E. corrodens* and* S. oralis*, and bacterial adhesion was more significant in those made of Bioplast and Teflon. The piercings presented bacterial colonies on their surface, being higher in plastic piercings probably due to their uneven and rough surface.

## 1. Introduction

The use of oral piercing, including mouth and lips, can cause serious systemic health problems, regardless of being in soft or hard tissues, in the case of individuals with compromised immune systems. Among numerous piercing locations in the oral cavity, lip and tongue piercings stand out, the latter being one of the most prevalent [1].* Streptococcus oralis* and* Eikenella corrodens* are among the main bacteria that adhere to the surface of tongue piercings [2].

It is also known that some material properties may interfere with microbial colonization on solid surfaces such as chemical composition, surface roughness, cracks and inclusions, coverage by oxide films or organic coatings, and properties inherent in the material used [3]. Piercings are made of different materials, most frequently metals such as surgical steel and titanium [4, 5]. Recently, piercings have also been made of synthetic materials such as Teflon, nylon, and plastic [6].

The most commonly used types of piercing are those with the shape of a rod [7]. The most common is the one with a ball-shaped end (94% of cases) followed by piercing with cone-shaped end (4% of cases) and those with a cylinder-shaped end are the least popular, with only 2% of the cases [6]. There are various complications associated with the use of oral piercing, which can compromise oral health and lead to periodontal problems and tooth injuries [8]. These include pain, swelling, infection, disease transmission, obstruction of the secondary airway due to bleeding, prolonged bleeding, chipped or fractured teeth, trauma to the gingival mucosa, interference with chewing or salivation, speech problems, drooling, formation of hyperplastic or scarring tissue, nerve damage and paresthesia, artifact aspiration, incorporation of foreign body into the piercing site, distortion of radiographic images, calculus formation on metal surfaces, and hypersensitivity to the metal [9, 10].

A greater amount of biofilm can be produced at the oral piercing site due to the difficulty of maintaining local hygiene and food debris retention, creating an ideal environment for large accumulation of plaque and calculus. In addition, the constant contact with the object increases the likelihood of appearance of supra- and subgingival biofilm in lower central incisors. These accumulations can produce halitosis and possible infection [10, 11].

The aim of this study was to investigate the* in vitro* adherence of* Eikenella corrodens* and* Streptococcus oralis* to the surface of tongue piercings made of surgical steel, titanium, polypropylene (Bioplast), and polytetrafluoroethylene (Teflon), quantifying CFU/mL adhered to these materials and describing, by means of scanning electron microscopy, the characteristics of this colonization.

## 2. Material and Methods

### 2.1. Selection of Strains and Piercings

*Streptococcus oralis* (ATCC 10557) and* Eikenella corrodens* (ATCC 23834) were used as test organisms. Such microorganisms were provided by the National Institute for Quality Control in Health (INCQS), FIOCRUZ (Oswaldo Cruz Foundation), located in the city of Rio de Janeiro/RJ, Brazil. Tongue piercings made of surgical steel, titanium, Bioplast, and Teflon were selected, all of the same brand and with the shape of rod with two balls at each end. Thus, the sample consisted of 192 tongue piercings. For each material, 40 piercings were intended for microbiological testing and another 8 for SEM analysis. Samples were randomly selected at the time of purchase. The inclusion criteria for each type of piercing were that the materials were made by the same manufacturer and had the same preestablished size and shape.

### 2.2. Preparation of Bacterial Inoculum

Test strain suspensions were prepared in sterile saline, which were standardized according to the 0.5 tube of the McFarland Nephelometric Scale (PROBAC DO BRASIL®, São Paulo, SP, Brazil) corresponding to the concentration of about 10^8^ Colony Forming Units per milliliter (CFU/mL).

### 2.3. Assemblage of* In Vitro* Adhesion Systems

Samples were divided into two groups according to the type of solution, where each group had 96 previously autoclaved piercings, 24 of each type. Of these, 20 of each type were used for bacterial adhesion test and 4 for SEM analysis. The first group (S1) showed 96 test tubes containing 5 mL of BHI broth (Brain Heart Infusion, HIMEDIA®, Mumbai, India) and 50 *μ*L of bacterial inoculum and the second group (S2) had 96 test tubes containing 5 mL BHI broth serving as sterility control of the culture medium and samples. After placement of piercing with the aid of sterile tweezers, the tubes were incubated in bacteriological incubator at 37°C in microaerophilic conditions for a period of 24 hours.

### 2.4. Bacterial Adhesion Analysis

After 24 hours, each piercing was transferred with the aid of a platinum loop flamed until red to a test tube containing 5 mL of sterile saline, being then stirred for 2 minutes on AP 59 solution stirrer (Phoenix®, Araraquara, SP, Brazil) at speed 5. Then, the solution obtained was serially diluted in sterile saline to 10^−5^. Thus, for each sample, five test tubes containing 4.9 mL of sterile saline were prepared. Then, serial dilution was performed. Aliquots of 25 *μ*L of 10^−1^, 10^−2^, 10^−3^, 10^−4^, and 10^−5^ dilutions were inoculated into dishes containing BHI (Brain Heart Infusion) culture (DIFCO®, São Paulo, SP, Brazil) using the drop technique [12]. For each piercing, two Petri dishes divided into three equal parts were used. In each part of the dish, 3 aliquots of 25 *μ*L of each dilution were inoculated. Dishes were incubated in a bacteriological incubator at 37°C for 48 hours in microaerophilic conditions for later counting of Colony Forming Units (CFU/mL). For each sample, the count of three drops of dilution that showed the lowest number of colonies was performed. The average of the three counts was multiplied by 40 and raised to the power of the corresponding dilution in order to estimate the number of CFU/mL, that is, CFU/piercing [13].

### 2.5. Scanning Electron Microscopy Analysis

After the incubation period (24 hours), the 32 piercings were prepared for SEM analysis. Then, the piercings were mounted on aluminum sample holder and covered with a thin layer of gold through the Emitech K550X sputter machine (Emitech®, Molfetta, Italy). After coating, the samples were analyzed and photographed under SEM (LEO 1430®, Elektronen Mikroskopie GmbH, UK) with magnifications of 100x and 500x. This analysis enabled observing the surface of piercings and the organization of bacterial colonization on these surfaces.

### 2.6. Data Analysis

Data on the count of CFU/mL were tested for normality by Kolmogorov-Smirnov test. Significant statistical differences of the mean values (CFU/mL) of* E. corrodens *and* S. oralis *among the four types of piercings were examined using Kruskal-Wallis test. Student's *t*- and Mann-Whitney *U* tests were used to compare each material in relation to both bacteria. The piercing types were compared two-by-two for the appropriate test according to the normality (Student's *t-*test for continuous normally distributed and Mann-Whitney *U* test for continuous skewed variables). For this, the Statistical Package for Social Sciences (SPSS) software version 20 was used. Data on SEM analysis were descriptively analyzed, showing the surface of the four types of piercings and respective bacterial colonization.

## 3. Results

In the control group S2 (incubation in BHI only), bacterial adherence was not observed on the surface of any type of piercing, confirming the sterility of samples and culture medium used.

The CFU count of each bacterium and piercing is shown in Table 1. It was observed that there was adhesion of both microorganisms in all piercings, and those made of titanium were the least adhered with 6.40 × 10^5^ CFU/mL for* E. corrodens* and 24.21 × 10^5^ CFU/mL for* S. oralis*. The piercings of Bioplast were the most adhered of* E. corrodens* (67.36 × 10^5^ CFU/mL) and* S. oralis* (77.38 × 10^5^ CFU/mL). There was also a statistically significant difference (*p* < 0.05) between materials tested on the same bacteria. A statistically significant difference was observed when each material was compared in relation to both bacteria, and only for Bioplast this difference was not observed (*p* > 0.05) (Table 1).

When comparing the different materials two-by-two (Mann-Whitney *U* test) for* E. corrodens*, it was found that, except to Bioplast compared to Teflon (*p* = 0.089), the other types of piercings showed statistically significant difference between them (*p* < 0.05). For* S. oralis*, the comparison of two different materials two-by-two (Mann-Whitney *U* test) indicated there was no statistically significant difference when surgical steel was compared with titanium (*p* = 0.353) and Teflon (*p* = 0.063) and also when Bioplast and Teflon are compared (*p* = 0.631).

**Figure 1:**
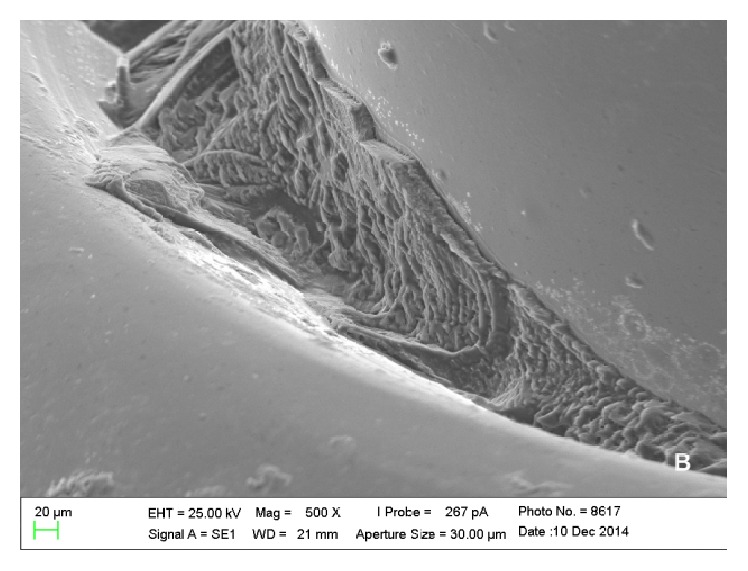
Electronic micrograph of the surface of a surgical steel piercing incubated in solution containing* S. oralis* (interface between the ball and the rod at 500x magnification).

**Figure 2:**
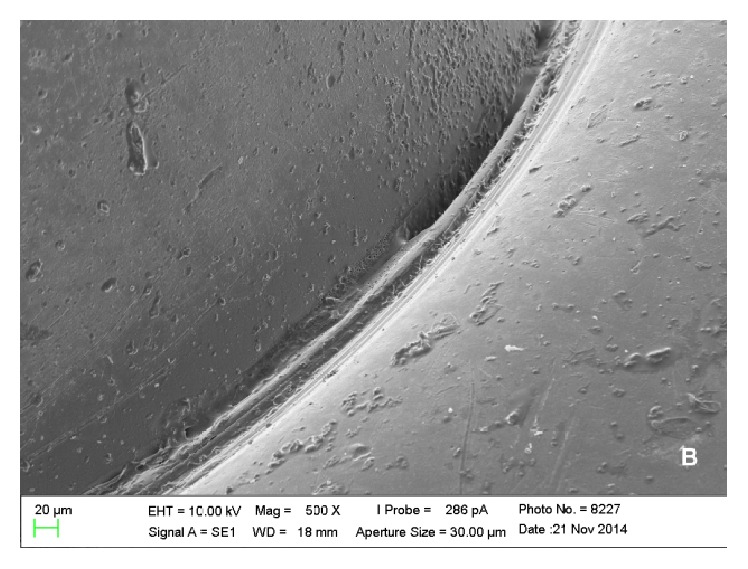
Electronic micrograph of the surface of a titanium piercing incubated in solution containing* E. corrodens* (interface between the ball and the rod at 500x magnification).

**Figure 3:**
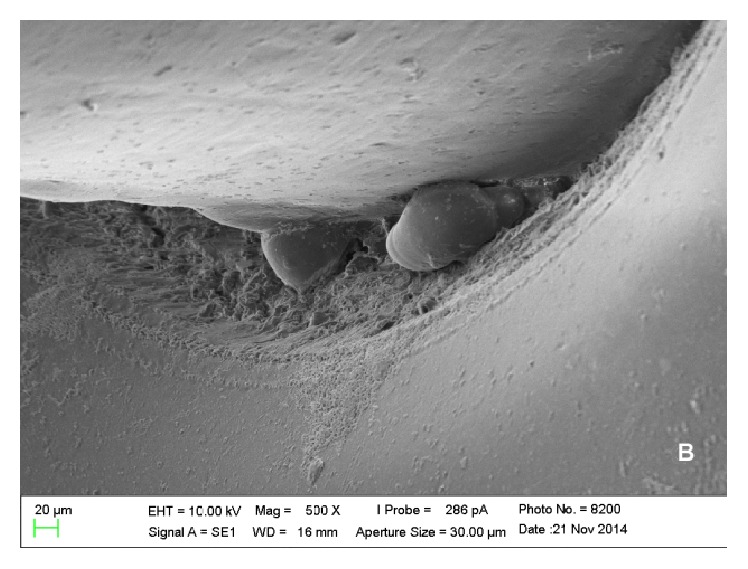
Electronic micrograph of the surface of a Bioplast piercing incubated in solution containing* E. corrodens* (interface between the ball and the rod at 500x magnification).

**Figure 4:**
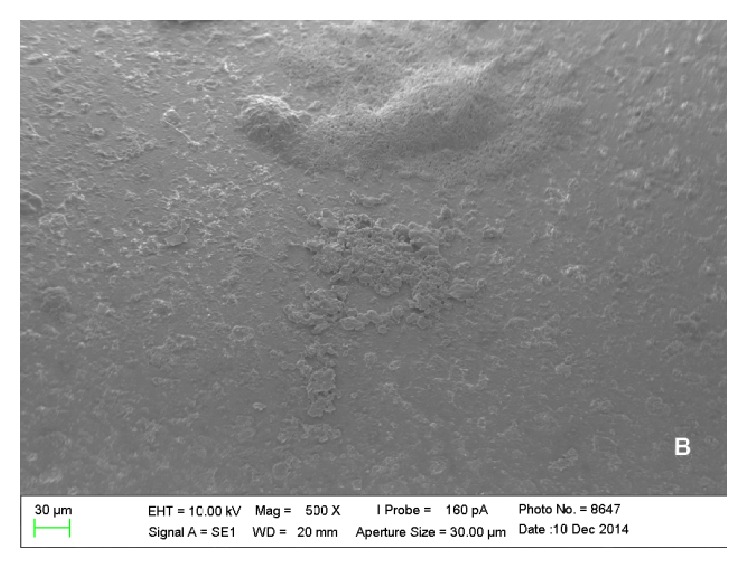
Electronic micrograph of the surface of a Teflon piercing incubated in solution containing* S. oralis* (ball at 500x magnification).

Scanning electron microscopy analysis showed colonies of* E. corrodens *and* S. oralis *on the surfaces of piercings, which could be viewed scattered and in lower amounts in metal piercings (Figures 1 and 2) when compared with plastic piercings (Figures 3 and 4). Data did not show microorganisms on the surface of piercings incubated in BHI (control).

**Figure 5:**
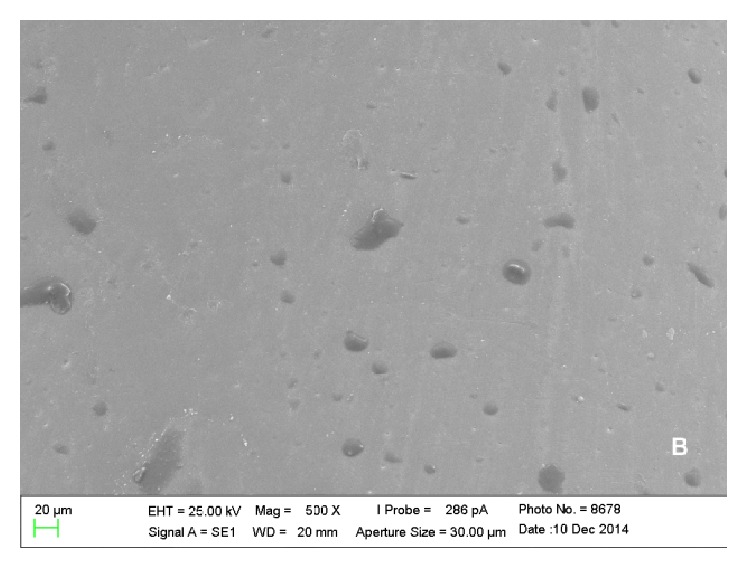
Electronic micrograph of the surface of a titanium piercing incubated in solution containing only BHI (500x magnification) presenting smooth and polished surface.

**Figure 6:**
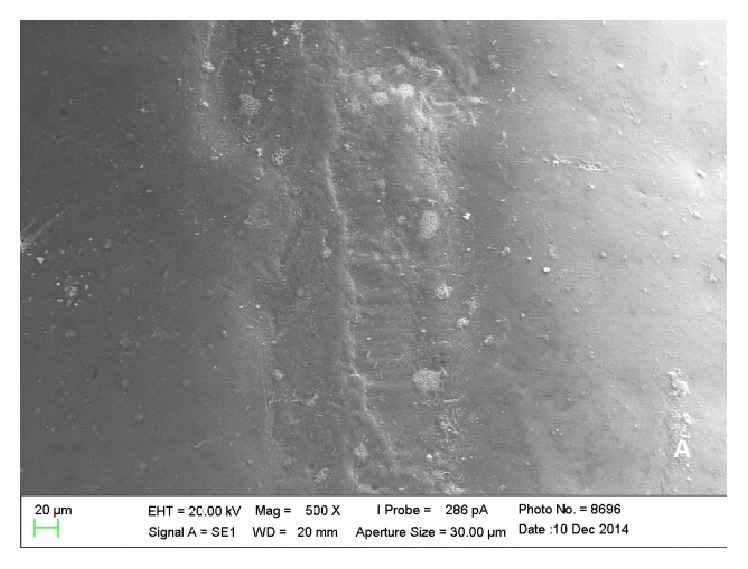
Electronic micrograph of the surface of a Bioplast piercing incubated in solution containing only BHI (500x magnification) presenting a rough and uneven surface.

Furthermore, the analysis allowed understanding of the spatial configuration of piercings and the detailed viewing of their surfaces. Thus, it was observed that metal and plastic piercings have different shapes and surfaces. The former have smooth and polished surface (Figure 5), while plastic piercings have rough and uneven surface (Figure 6).

## 4. Discussion

In this study,* E. corrodens* and* S. oralis* strains were used with the aim of facilitating the understanding of data and evaluating how each bacterium behaves separately in the presence of piercings tested. However, it is known that both* in vivo* bacteria have reciprocal action between themselves and with a wide range of other bacteria, and thus it is interesting to conduct studies on the adhesion to piercings in a microbial biofilm model.

*Eikenella corrodens* and* Streptococcus oralis* were the bacteria chosen to be tested because the study by Kapferer et al. [2] demonstrated a significant presence of these bacteria on the surface of tongue piercings.* Eikenella corrodens* composes a short list of likely candidates as periodontal pathogens [14] and is associated with chronic osteomyelitis of the jaws [15]. Bacteriological studies revealed that Gram-positive bacteria, including* S. oralis*, are early colonizers in oral biofilm [16] and are species relatively abundant and prevalent in periodontitis and peri-implantitis [17].

The choice of piercing materials that would be used in the experiment was due to the fact that several studies showed that surgical steel, titanium, Bioplast, and Teflon piercings are the most commonly found in patients and are easily accessible to the general population [4, 5, 18].

There are some studies in literature involving individuals with tongue piercing [1, 18–21]. These studies only address aspects such as prevalence of complications from the use of tongue piercing and its influence on periodontal diseases. Only two studies included the microbiology related to the use of tongue piercing [2, 6]. However, these studies were conducted* in vivo* and there are no studies done* in vitro* to analyze tongue piercing. In the first study, Kapferer et al. [2] collected microbiological samples of 85 subjects with tongue piercings 2 weeks after four different materials were randomly allocated among them. It was observed that 84% and 35% of the bacterial species were found at significantly higher levels (*p* < 0.001), respectively, in samples from stainless steel and titanium than from plastics piercings. The second research [6] involved 12 patients with tongue piercings with the purpose to investigate the presence of 11 periodontopathogenic bacteria in these sites. The microbiological analysis showed an increased or substantially increased concentration of periodontopathogenic bacteria in all subjects. The authors affirm that it is questionable whether the material of the piercing may play an additional role in plaque accumulation.

Oral piercing made of surgical steel showed good results with respect to adhesion for both* E. corrodens* and* S. oralis*, but according to Espírito Santo et al. [22] this material may be associated with oral cancer due to chrome release, considered a carcinogenic substance. Bordji et al. [23] had already warned about metal alloys that have chrome in their composition. It was found that, in studies in human tissues, chrome has the ability to concentrate in the nucleus and in the mitochondria of cells, inhibiting oxidative metabolism, interacting with DNA and RNA, and inducing the formation of neoplastic cells [24].

Table 1 shows that metal piercings present less bacterial adherence when compared with plastic piercings. This result differs from the findings of Kapferer et al. [2] who found that stainless steel piercings had higher bacterial adhesion, while plastic piercings had become inert to colonization. These controversial results could be due to differences in experimental conditions; in particular, laboratory models cannot completely reproduce the biodiversity and heterogeneity of natural dental biofilms; the present study used reference strains.

Roughness is a determining factor for bacterial adherence, but there are physical and chemical factors as well as the presence of other microorganisms* in vivo* medium that can influence bacterial adhesion, as inter- and intraspecies coaggregation and coadhesion interactions [25–27]. Based on this, another hypothesis for the differences between these two studies might be related to the manufacturing processes. Since the resulting surface structure depends on this process, it is possible that piercings made with the same material, for example, metal or plastic, but from different commercial brands, have dissimilar mechanical irregularities and divergent resistance to corrosion [28].

The dynamism of the oral environment, together with the host's immunological mechanisms, environmental conditions, microbiota microorganisms, especially diet, and oral hygiene cannot be reproduced in laboratory environment [29, 30]. As* in vitro* studies provide a simplified view of the medium, it is important to use* in vivo* models suitable to validate* in vitro* results as the first step to test hypotheses that could be translated into larger organisms or clinical trials [31].

The use of scanning electron microscopy in the analysis of samples is a method that can be considered as the gold standard in the analysis of a surface, as it is probably the most sensitive means to verify small structural changes [32]. Several studies have used SEM to evaluate the adherence of bacteria to teeth or different dental materials [33–35]. However, there are no studies in literature analyzing the surface of tongue piercings by scanning electron microscope or the adhesion of bacteria to these artifacts.

In the present experiment, it could be concluded that there are differences between metal and plastic piercings in relation to* Eikenella corrodens* and* Streptococcus oralis* colonization by SEM analysis. Metal piercings have polished and smooth surface, but plastic ones showed rough and uneven surface. Regarding the influence of roughness on biofilm formation, rougher surfaces result in increased bacterial adhesion [26, 27].

This fact may be responsible for the difference observed in the microbiological step between the two types of tongue piercings. Consequently, the amount of bacterial colonies of both* E. corrodens* and* S. oralis* adhered to piercings was significantly smaller in metal piercings compared to plastic piercings.

Given the growth of people that make use of piercings, especially tongue piercings, the user should have access to information on which material used is the most harmful to the oral health, as the use of materials less susceptible to biofilm accumulation can contribute to the prevention of oral diseases. The patient should be guided not to use tongue piercing, as it can bring many harms, but if the patient insists, piercings made of titanium are the most suitable, since, according to the study by Carlsson et al. [36], titanium piercings are those with less corrosion and higher biocompatibility and according to Ziebolz et al. [18], they do not present chromium release, such as surgical steel piercings. Furthermore, in this study, titanium piercings have shown less bacterial adherence of both bacteria tested.

Thus, there is need for further studies on this topic in order to have a better understanding of which materials used in the manufacture of tongue piercings are more suitable, thereby reducing the risk of infection and complications that may arise from the use of this artifact.

## 5. Conclusion

The four types of piercings seemed to be susceptible to colonization by* E. corrodens* and* S. oralis*, and bacterial adhesion was more significant in piercings made of Bioplast and Teflon. Scanning electron microscopy showed that the four types of piercings presented bacterial colonies on their surface, with greater amounts found in plastic piercings probably due to their uneven and rough surface.

## Figures and Tables

**Table 1:** Mean adherence of *E. corrodens* and *S. oralis* in CFU/mL for the four types of piercings.

	Surgical steel	Titanium	Bioplast	Teflon	*p* value^**∗**^
*E. corrodens*	11.95 × 10^5^	6.40 × 10^5^	67.36 × 10^5^	36.72 × 10^5^	**0.001**
*S. oralis*	29.68 × 10^5^	24.21 × 10^5^	77.38 × 10^5^	68.34 × 10^5^	**0.007**
*p* value^**∗**^	0.002^*∗∗*^	0.010^*∗∗∗*^	0.666^*∗∗∗*^	0.038^*∗∗∗*^	

Test used: Kruskal-Wallis^*∗*^; Mann-Whitney^*∗∗*^; *t*-test^*∗∗∗*^.
